# Association between metabolic overweight/obesity phenotypes and readmission risk in patients with Immune Thrombocytopenia (ITP): a national retrospective study

**DOI:** 10.1007/s00277-026-07182-y

**Published:** 2026-07-23

**Authors:** Fuxin Xu, Tongxin Wang, Lingquan Meng, Cheng Zhang, Xin Zhou, Xiangxiang Zhou, Panpan Han

**Affiliations:** 1 Department of Hematology, Shandong Provincial Hospital Affiliated to Shandong First Medical University, No. 324, Jingwu Road, Jinan, Shandong 250021 China; 2 Department of Hematology, Shandong Provincial Hospital, Shandong University, Jinan, Shandong 250021 China; 3 Department of Endocrinology, Shandong Provincial Hospital Affiliated to Shandong First Medical University, Jinan, Shandong 250021 China; 4 Department of Urology, The First Affiliated Hospital of Shandong First Medical University, Shandong Provincial Qianfoshan Hospital, Jinan, China

**Keywords:** ITP, Immune thrombocytopenia, Metabolic disorders, Overweight/obesity phenotypes, Readmission

## Abstract

Immune thrombocytopenia (ITP) is one of the most common autoimmune hemorrhagic disorders and is characterized by frequent relapses that require repeated hospitalization. Although overweight/obesity and metabolic disorders have been linked to ITP, their association with readmission risk remains unclear. A cohort of 3,811 patients with a discharge diagnosis of ITP was identified from the National Readmission Database (NRD) for this retrospective analysis. Patients were classified into four phenotypes according to overweight/obesity status and metabolic status: metabolically healthy non-obese (MHNO), metabolically unhealthy non-obese (MUNO), metabolically healthy obese (MHO), and metabolically unhealthy obese (MUO). Readmissions were assessed at 30, 60, 90, 120, 150, and 180 days. Multivariable Cox proportional hazards models were used to evaluate the association between metabolic overweight/obesity phenotypes and readmission risk in this cohort. At 120, 150, and 180 days, patients with metabolically unhealthy phenotypes had a significantly higher risk of readmission than those with the MHNO phenotype. In contrast, the MHO phenotype was not associated with increased readmission risk. Subsequent stratified analyses showed a gradual increase in readmission risk with a higher number of concurrent metabolic risk factors. Among patients with ITP, metabolic disorders, rather than overweight/obesity, were associated with an increased risk of readmission after multivariable adjustment. Readmission risk also increased with the number of concurrent metabolic risk factors. Notably, the association between metabolic comorbidities and ITP-related readmission showed a distinct time-dependent pattern. Because this study did not include a non-ITP control group, whether this association is specific to ITP or reflects a broader effect of metabolic comorbidities on readmission remains uncertain.

**Supplementary Information:**

The online version contains supplementary material available at https://doi.org/10.1007/s00277-026-07182-y.

## Introduction

 Immune thrombocytopenia (ITP) is the most prevalent autoimmune hemorrhagic disorder encountered in hematological practice. Its pathogenesis is complex and highly heterogeneous. ITP is generally considered to arise from impaired immune tolerance and aberrant immune activation, resulting in increased platelet clearance and impaired platelet production [1, 2]. Clinically, ITP presents as thrombocytopenia with bleeding of variable severity, ranging from petechiae, ecchymoses, and mucosal bleeding to severe complications such as hematuria, hematochezia, and life-threatening intracranial hemorrhage [3].

Despite substantial therapeutic advances, ITP remains difficult to manage. Most affected patients experience relapse and require repeated hospitalization [4]. Hospital readmission is widely used as an indicator of healthcare utilization and quality of care and has also been associated with long-term clinical outcomes [5, 6]. In the context of initiatives such as the U.S. Hospital Readmission Reduction Program (HRRP), identifying factors that contribute to readmission may support more individualized post-discharge follow-up, reduce avoidable rehospitalization, and lessen healthcare burden [7]. For patients with ITP, recurrent admissions impose substantial strain on both healthcare resources and quality of life. Defining reliable predictors of readmission is therefore important for reducing the ongoing clinical and economic burden of this disease.

Obesity has become a major global public health concern and is a well-established risk factor for chronic conditions, including cardiovascular disease, diabetes, and several malignancies [8]. Increasing evidence also suggests that overweight/obesity and metabolic abnormalities may influence the onset and clinical course of autoimmune diseases, including ITP [9]. However, the specific relevance of these factors to ITP remains uncertain.

Few studies have evaluated whether metabolic overweight/obesity phenotypes are associated with readmission risk in patients with ITP. Clarifying this association may help identify high-risk subgroups that could benefit from targeted post-discharge management. Therefore, using the U.S. NRD, this study examined the association between distinct overweight/obesity and metabolic phenotypes and readmission risk among adults with ITP. We aimed to characterize readmission patterns by metabolic overweight/obesity phenotype and to provide real-world observational evidence for post-discharge risk stratification, while recognizing that the observed associations may not be specific to ITP.

## Methods

### Data source

This retrospective study used the 2018 NRD, which is part of the Healthcare Cost and Utilization Project (HCUP) sponsored by the Agency for Healthcare Research and Quality (AHRQ). The NRD is a longitudinal administrative database that enables patient-level tracking of hospitalizations within a single calendar year. The 2018 NRD includes data from 28 states and represents approximately 60% of the U.S. population and 58.7% of all U.S. hospitalizations [10]. Long-term care hospitals and rehabilitation facilities are not included in the NRD.

### Study population

**Fig. 1:**
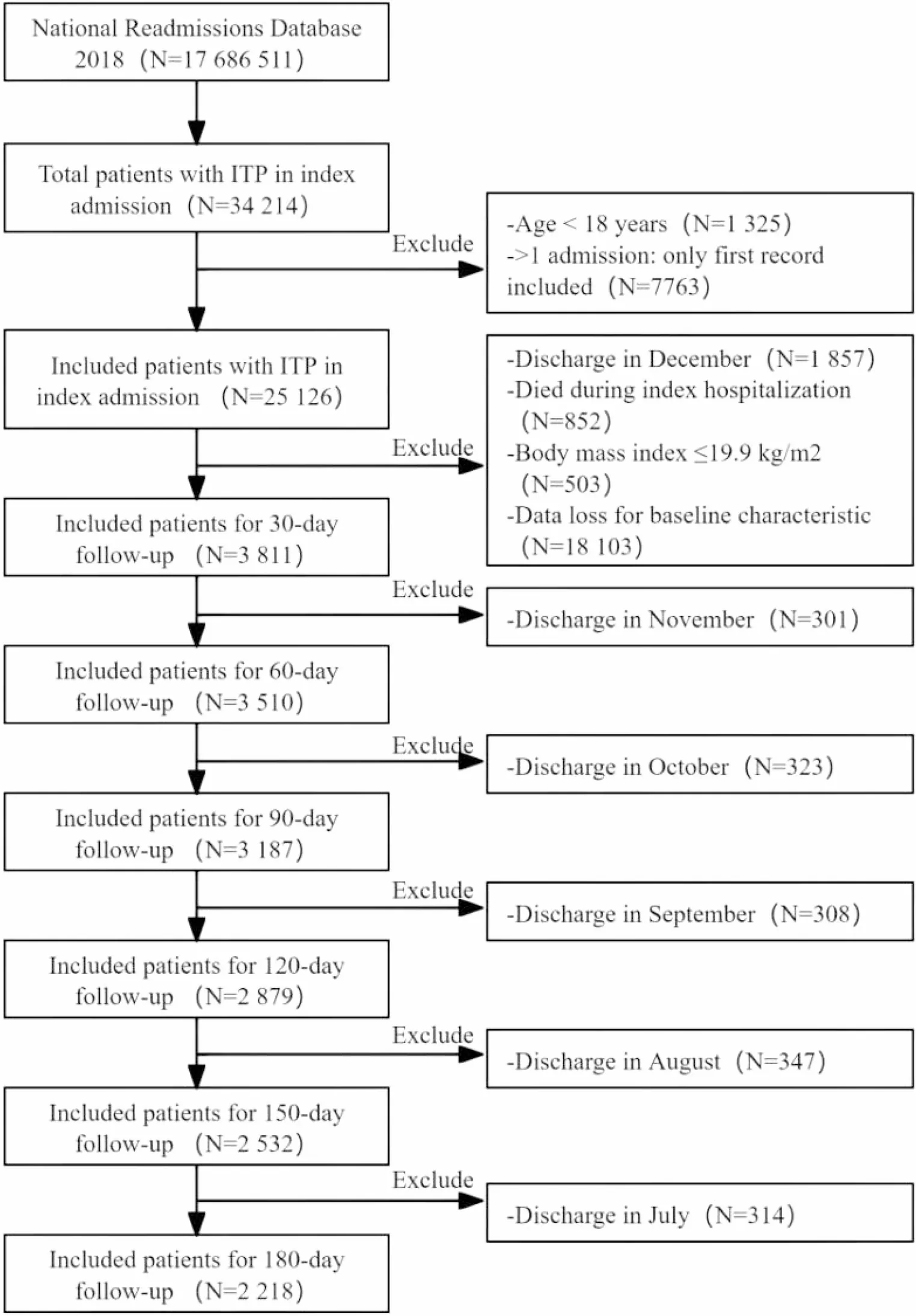
Flow chart of data screening

Adult patients aged 18 years or older who had a discharge diagnosis of ITP in 2018 were identified using the International Classification of Diseases, 10th Revision, Clinical Modification (ICD-10-CM) (Supplementary Table 4). The principal diagnosis code used for ITP was D69.3. Patients were excluded if key hospitalization data were missing, if they died during the index hospitalization, if they were younger than 18 years, or if they had low body weight, defined as a body mass index (BMI) ≤ 19.9 kg/m². Because of the inherent limitations of the NRD, information on ITP disease phase, disease severity, and treatment regimens was unavailable. For patients with more than one readmission, only the first recorded readmission was included to avoid overestimating readmission risk. Because the NRD does not allow follow-up beyond the calendar year, patients discharged in the later months of 2018 were excluded according to the required follow-up window. Specifically, discharges in December were excluded for the 30-day analysis; discharges in November and December for the 60-day analysis; discharges from October through December for the 90-day analysis; discharges from September through December for the 120-day analysis; discharges from August through December for the 150-day analysis; and discharges from July through December for the 180-day analysis (Fig. 1).

### Patient characteristics

Baseline demographic and hospitalization characteristics were extracted for each patient. These variables included age, gender, length of stay (LOS), primary payer, hospital location, discharge disposition, tobacco use, alcohol use, transfer to rehabilitation care, elective admission status, total charges, and comorbidities. Primary payer was categorized as Medicare, Medicaid, private insurance, self-pay, charity care, or other.

### Definitions

Readmission was defined as any hospital readmission for any diagnosis within 30, 60, 90, 120, 150, or 180 days after discharge from the index hospitalization. When multiple readmissions occurred, only the first readmission was counted. In the primary analysis, overweight/obesity was defined as BMI ≥ 25 kg/m² [9]. BMI was further categorized as overweight, 25.0–29.9 kg/m²; class I obesity, 30.0–34.9 kg/m²; class II obesity, 35.0–39.9 kg/m²; and class III obesity, ≥ 40 kg/m². To evaluate the robustness of the main findings under an alternative BMI threshold, a sensitivity analysis was conducted in which obesity was redefined as BMI ≥ 30 kg/m². The core statistical models, covariate adjustments, and outcome definitions were otherwise kept consistent with the primary analysis. Full sensitivity results, including readmission risk, age- and sex-stratified analyses, and hospitalization burden (Supplementary Text 1 and Supplementary Tables 1–3).

No universally accepted definition of metabolic health is currently available. In previous studies, metabolically unhealthy status has commonly been defined as the presence of two or more metabolic risk factors. Based on the criteria of the Third Report of the National Cholesterol Education Program Adult Treatment Panel III (NCEP ATP-III) and the consensus recommendations of the International Diabetes Federation (IDF) [11, 12], metabolic risk factors in this study included hypertension, dyslipidemia, and hyperglycemia. Hypertension was defined as primary hypertension, secondary hypertension, or undiagnosed elevated blood pressure. Dyslipidemia was defined as elevated serum triglyceride (TG) levels, reduced high-density lipoprotein (HDL) cholesterol levels, or other related lipid abnormalities. Hyperglycemia was defined as prediabetes, diabetes mellitus, or other specific types of diabetes. Abdominal obesity was not included as a metabolic risk factor because of collinearity with BMI. Metabolically unhealthy status was defined as the presence of at least two of these metabolic risk factors.

Individuals were classified into four phenotypes according to overweight/obesity and metabolic status [13]: metabolically healthy non-obese (MHNO), metabolically unhealthy non-obese (MUNO), metabolically healthy obese (MHO), and metabolically unhealthy obese (MUO). They were also grouped according to the type of concomitant metabolic risk factor: no metabolic risk factors, isolated hyperglycemia, isolated dyslipidemia, or isolated hypertension. In addition, participants were stratified by the number of concurrent metabolic risk factors as follows: no metabolic risk factors, one metabolic risk factor, two metabolic risk factors, or three metabolic risk factors.

### Outcomes

The primary outcome was all-cause hospital readmission across six predefined follow-up intervals among patients with ITP, stratified by metabolic overweight/obesity phenotype. Secondary outcomes included LOS, mean total hospital charges, and readmission mortality.

To further characterize the clinical context of readmission, the principal discharge diagnosis recorded during the first readmission hospitalization was extracted for each follow-up interval. Diagnoses were summarized using ICD-10-CM codes and corresponding diagnostic categories (Supplementary Table 4). For descriptive analyses, diagnoses were reported if they accounted for at least 1% of readmission events or ranked among the 10 most common principal readmission diagnoses at each follow-up interval. Because ITP may be recorded as a secondary rather than principal readmission diagnosis, the cumulative proportion of readmitted patients with ITP listed among the top 10 discharge diagnoses was also summarized where available.

### Statistical analysis

Descriptive statistics were used to summarize baseline demographic and admission-level characteristics across metabolic overweight/obesity phenotypes. Categorical variables were compared using the χ² test and are presented as counts and percentages. Continuous variables were compared using analysis of variance (ANOVA) and are reported as means ± standard deviations (SDs). Unadjusted hazard ratios (HRs) were estimated using univariate Cox proportional hazards models. Adjusted hazard ratios (aHRs) were estimated using multivariable Cox regression models that included potential confounders. Based on prior literature and univariate analyses, the adjusted models included age, sex, elective admission, primary payer, household income, discharge disposition, LOS, total charges, emergency department records, transfer to rehabilitation care facilities, treatment location, local resident status, tobacco use, alcohol abuse, personal history of cancer chemotherapy, and obesity classification. All tests were two-sided, and *P* < 0.05 was considered statistically significant. All statistical analyses were performed using SPSS software (version 27.0). Before constructing the multivariable Cox proportional hazards regression models, the proportional hazards (PH) assumption was evaluated for all covariates and exposure groups using Schoenfeld residual analysis. No significant time-dependent interaction was detected (all *P* > 0.05), indicating that the PH assumption was satisfied for all variables included in the regression framework. To reduce confounding bias, the multivariable Cox regression models incorporated a comprehensive set of demographic, hospitalization-related, behavioral, and comorbidity covariates. However, because of the inherent limitations of administrative billing databases, residual confounding could not be fully eliminated, as granular clinical variables that may independently predict ITP readmission risk were unavailable.

## Results

### Patient baseline characteristics

Among 17,686,511 discharge records in the 2018 Nationwide Readmissions Database, 3,811 patients with a diagnosis of ITP met the inclusion criteria. The numbers of participants included in the 30-, 60-, 90-, 120-, 150-, and 180-day follow-up analyses were 3,811, 3,510, 3,187, 2,879, 2,532, and 2,218, respectively.

The mean age of the enrolled patients was 59.7 years, and 38.2% were aged 50–70 years. Most patients were female (60.1%) and covered by Medicare (53.1%). Overall, 3,478 patients (91.3%) were overweight or obese, and 1,955 patients (51.3%) were metabolically unhealthy.

Across the four phenotypes, the MUNO group had the highest mean age and the longest LOS (12.1 days), whereas the MHNO group had the highest mean total hospital charges (USD 152,854.6). Compared with the MHNO and MHO groups, the MUNO and MUO groups included a higher proportion of elderly patients aged ≥ 70 years and had higher prevalence rates of heart failure, renal failure, and coronary heart disease. Baseline demographic and clinical characteristics are presented in Table 1.

**Table 1:** Baseline clinical and demographic characteristics of index hospitalizations for patients

Variables	MHNO	MUNO	MHO	MUO	Total	*P* value
**No of cases**	201(5.3%)	132(3.5%)	1655(43.4%)	1823 (47.8%)	3811	
**Age(years)(mean(SD))**	63.9(18.8)^a^	**76.0(11.6)** ^**b**^	51.0(17.7)^c^	65.9(12.5)^a^	59.7(17.2)	**< 0.001**
**Age groups**						**< 0.001**
≤ 50 years	45(22.4%)^a^	5(3.8%)^b^	**815(49.2%)** ^**c**^	218(12.0%)^d^	1083(28.4%)	
50–70 years	71(35.3%)^a, b^	26(19.7%)^c^	545(32.9%)^b^	**814(44.7%)** ^**a**^	1456(38.2%)	
≥ 70years	85(42.3%)^a^	**101(76.5%)** ^**b**^	295(17.8%)^c^	791(43.4%)^a^	1272(33.4%)	
**Male**	109(54.2%)^a^	**75(56.8%)** ^**a**^	512(30.9%)^b^	824(45.2%)^a^	1520(39.9%)	**< 0.001**
**Primary payer**						**< 0.001**
Medicare	125(62.2%)^a^	**114(86.4%)** ^**b**^	572(34.6%)^c^	1212(66.5%)^a^	2023(53.1%)	
Medicaid	28(13.9%)^a, b^	7(5.3%)^b, c^	**331(20.0%)** ^**a**^	144(7.9%)^c^	510(13.4%)	
Private insurance	38(18.9%)^a^	10(7.6%)^b^	**640(38.7%)** ^**c**^	407(22.3%)^a^	847(28.7%)	
Self-pay	4(2.0%)^a, b^	1(0.8%)^a, b^	**63(3.8%)** ^**b**^	27(1.5%)^a^	95(2.5%)	
Free	3(1.5%)^a^	0(0.0%)^a^	8(0.5%)^a^	7(0.4%)^a^	18(0.5%)	
Other	3(1.5%)^a^	0(0.0%)^a^	41(2.5%)^a^	26(1.4%)^a^	70(1.8%)	
**Disposition**						**< 0.001**
Routine	96(47.8%)^a^	35(26.5%)^b^	**1228(74.2%)** ^**c**^	1076(59.0%)^d^	2435(63.9%)	
Short-term Hospital	2(1.0%)^a^	1(0.8%)^a^	18(1.1%)^a^	20(1.1%)^a^	41(1.1%)	
Skilled Nursing/Other Facility	48(23.9%)^a^	**53(40.2%)** ^**b**^	178(10.8%)^c^	323(17.7%)^a^	602(15.8%)	
Home Health Care	51(25.4%)^a, b^	**43(32.6%)** ^**b**^	221(13.4%)^c^	393(21.6%)^a^	708(18.6%)	
Against Medical Advice	4(2.0%)^a^	0(0.0%)^a^	10(0.6%)^a^	11(0.6%)^a^	25(0.7%)	
Discharge alive, destination unknown						
**Location**						**0.005**
Large central counties	64(31.8%)^a^	45(34.1%)^a^	457(27.6%)^a^	463(25.4%)^a^	1029( 27.0%)	
Large fringe counties	52(25.9%)^a^	44(33.3%)^a^	478(28.9%)^a^	495(27.2%)^a^	1069(28.1%)	
Medium metro counties	46(22.9%)^a^	20(15.2%)^a^	348(21.0%)^a^	376(20.6%)^a^	790(20.7%)	
Small metro counties	17(8.5%)^a^	6(4.5%)^a^	150(9.1%)^a^	206(11.3%)^a^	379(9.9%)	
Micropolitan counties	9(4.5%)^a^	8(6.1%)^a^	145(8.8%)^a^	158(8.7%)^a^	320(8.4%)	
Not metro/micropolitan counties	13(6.5%)^a^	9(6.8%)^a^	77(4.7%)^a^	125(6.9%)^a^	224(5.9%)	
**LOS(days)(mean(SD))**	10.2(12.6)^a^	**12.1(13.6)** ^**a**^	6.5(10.8)^b^	6.8(7.6)^b^	7.0(9.7)	**< 0.001**
**LOS > 3 days**	146(72.6%)^a^	**100(75.8%)** ^**a**^	842(50.9%)^b^	1067(58.5%)^c^	2155(56.5%)	**< 0.001**
**LOS > 7 days**	87(43.3%)^a^	**68(51.5%)** ^**a**^	358(21.6%)^b^	509(27.9%)^c^	1022(26.8%)	**< 0.001**
**Alcohol abuse**	**15(7.5%)** ^**a**^	8(6.1%)^a, b^	59(3.6%)^b^	46(2.5%)^b^	128(3.4%)	**0.002**
**Tobacco abuse**	**31(15.4%)** ^**a**^	9(6.8%)^a, b^	210(12.7%)^a^	155(8.5%)^b^	405(10.6%)	**< 0.001**
**Rehabtransfer**	1(0.5%)^a, b^	**7(5.3%)** ^**c**^	19(1.1%)^b^	49(2.7%)^a, c^	76(2.0%)	**< 0.001**
**Elective**	12(6.0%)^a^	10(7.6%)^a^	**333(20.1%)** ^**b**^	321(17.6%)^b^	676(17.7%)	**< 0.001**
**Total Charges($)(mean(SD))**	**152854.6 (297837.4)** ^**a**^	135667.8 (179071.3)^a, b^	92472.2 (149817.2)^c^	95292.0 (125285.4)^b, c^	98501.9 (152426.1)	**0.002**
**Total Charges** ≥ **$50,000**	124(61.7%)^a^	**82(62.1%)** ^**a**^	795(48.0%)^b^	968(53.1%)^a^	1969(51.7%)	**< 0.001**
**Comorbidities**						
Metabolically unhealthy	0(0.0%)^a^	132(100.0%)^b^	0(0.0%)^a^	1823(100.0%)^b^	1955(51.3%)	**< 0.001**
Obesity	0(0.0%)^a^	0(0.0%)^a^	1655(100.0%)^b^	1823(100.0%)^b^	3478(91.3%)	**< 0.001**
Hyperglycemia	10(5.0%)^a^	60(45.5%)^b^	135(8.2%)^a^	**1344(73.7%)** ^**c**^	1549(40.6%)	**< 0.001**
Dyslipidemia	13(6.5%)^a^	**111(84.1%)** ^**b**^	106(6.4%)^a^	1316(72.2%)^c^	1546(40.6%)	**< 0.001**
Hypertension	79(39.3%)^a^	**129(97.7%)** ^**b**^	635(38.4%)^a^	1728(94.8%)^b^	2571(67.5%)	**< 0.001**
Heart failure	29(14.4%)^a^	**44(33.3%)** ^**b**^	182(11.0%)^a^	581(31.9%)^b^	836(21.9%)	**< 0.001**
Renal failure	48(23.9%)^a^	**42(31.8%)** ^**a**^	214(12.9%)^b^	472(25.9%)^a^	776(20.4%)	**< 0.001**
Coronary heart disease	25(12.4%)^a^	**56(42.4%)** ^**b**^	119(7.2%)^a^	567(31.1%)^c^	767(20.1%)	**< 0.001**
Anemia	125(62.2%)^a^	**84(63.6%)** ^**a**^	637(38.5%)^b^	778(42.7%)^b^	1624(42.6%)	**< 0.001**
Neutropenia	6(3.0%)^a, b^	**5(3.8%)** ^**b**^	28(1.7%)^a, b^	18(1.0%)^a^	57(1.5%)	**0.021**
Depression	6(3.0%)^a^	2(1.5%)^a^	35(2.1%)^a^	42(2.3%)^a^	85(2.2%)	0.808

*LOS* length of stay; *MHNO* metabolically healthy nonobese; *MUNO* metabolically unhealthy nonobese; *MHO* metabolically healthy obese; *MUO* metabolically unhealthy obese. Values are No. (%) or as mean (SD) indicated. *P values were for the **χ²** test or the ANOVA across the four categories of overweight/obesity phenotypes. The small letters (e.g., a, b, c, d) in this table refer to comparisons between groups. There is no statistical difference between groups with the same small letters.

### Readmission risk

During the 30-, 60-, 90-, 120-, 150-, and 180-day follow-up periods, 427 (11.2%), 554 (15.8%), 574 (18.0%), 585 (20.3%), 549 (21.7%), and 524 (23.6%) patients were readmitted, respectively. Readmission rates for each metabolic overweight/obesity phenotype across the six follow-up intervals are presented in Table 2. Across all six follow-up cohorts, metabolically unhealthy phenotypes (MUNO and MUO) consistently showed higher readmission rates than metabolically healthy phenotypes (MHNO and MHO).

**Table 2:**
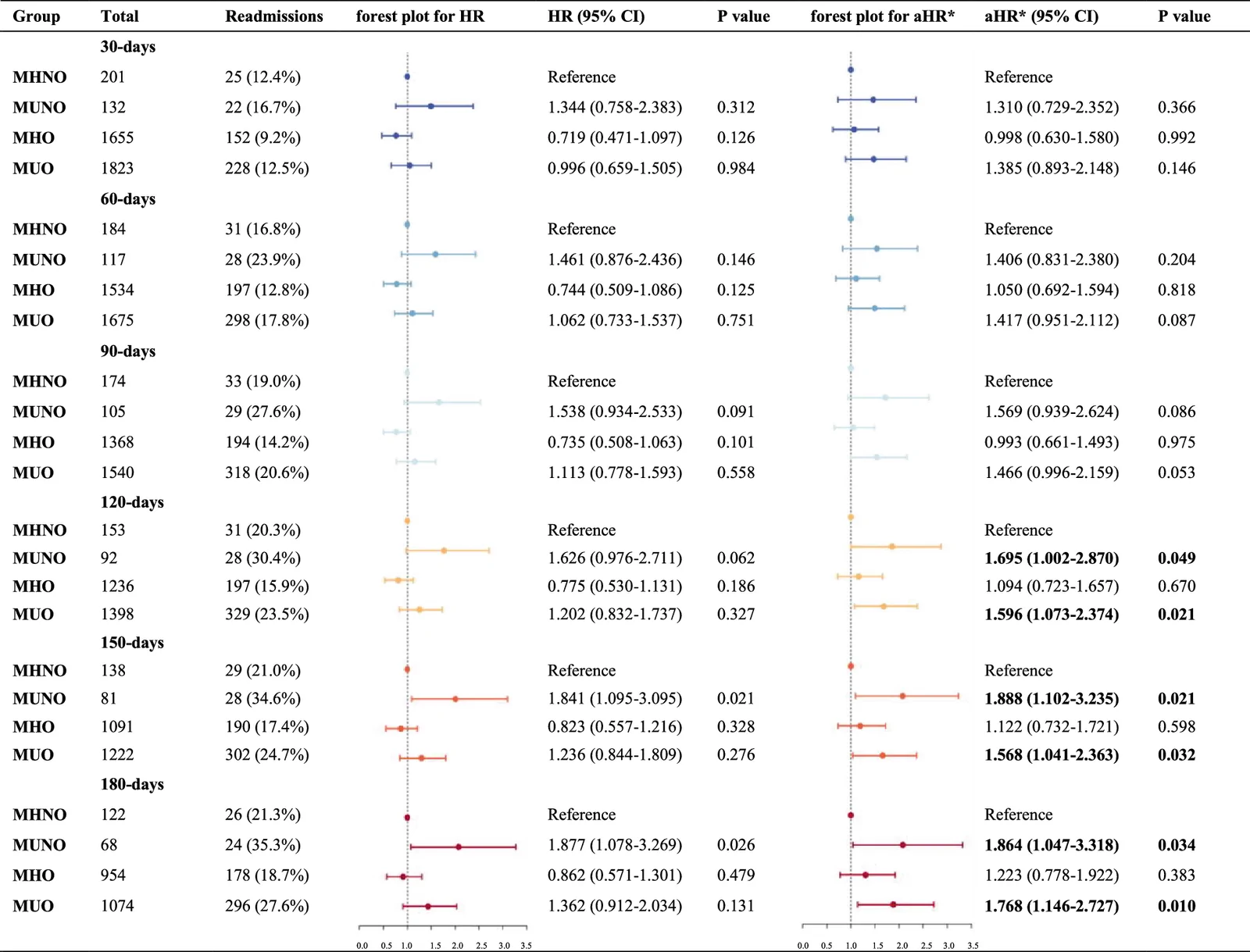
Readmission risk across six follow-up periods by different metabolic overweight/obesity phenotypes

The model was adjusted for age, gender, electiveadmission, primary payer, household income, patient discharge disposition,length of stay (LOS), total charges, emergency department records, transfer torehabilitation care facilities, patient treatment location, local residentstatus, tobacco use, alcohol abuse, personal history of cancer chemotherapy, andobesity classification

In the 30-, 60-, and 90-day analyses, readmission risk did not differ significantly among patients with different metabolic overweight/obesity phenotypes (Table 2).

At 120 days, however, both the MUNO group (aHR, 1.695; 95% CI, 1.002–2.870; *P* = 0.049) and the MUO group (aHR, 1.596; 95% CI, 1.073–2.374; *P* = 0.021) had a significantly higher risk of readmission than the MHNO group. Similar associations were observed at 150 days for MUNO (aHR, 1.888; 95% CI, 1.102–3.235; *P* = 0.021) and MUO (aHR, 1.568; 95% CI, 1.041–2.363; *P* = 0.032) relative to MHNO. At 180 days, the readmission risk remained higher in the MUNO group (aHR, 1.864; 95% CI, 1.047–3.318; *P* = 0.034) and the MUO group (aHR, 1.768; 95% CI, 1.146–2.727; *P* = 0.010) than in the MHNO group. In contrast, the MHO phenotype was not associated with increased readmission risk at any follow-up interval.

In analyses stratified by the type of metabolic risk factor (Table 3), patients with isolated hyperglycemia had a higher readmission risk at 90 and 120 days than those without metabolic risk factors (90 days: HR, 1.666; 95% CI, 1.090–2.548; *P* = 0.018; 120 days: HR, 1.588; 95% CI, 1.035–2.438; *P* = 0.034). After adjustment for potential confounders, this association remained significant in the 90-day analysis. By contrast, isolated dyslipidemia was associated with a consistently lower readmission risk across several follow-up intervals compared with no metabolic risk factors.

**Table 3:**
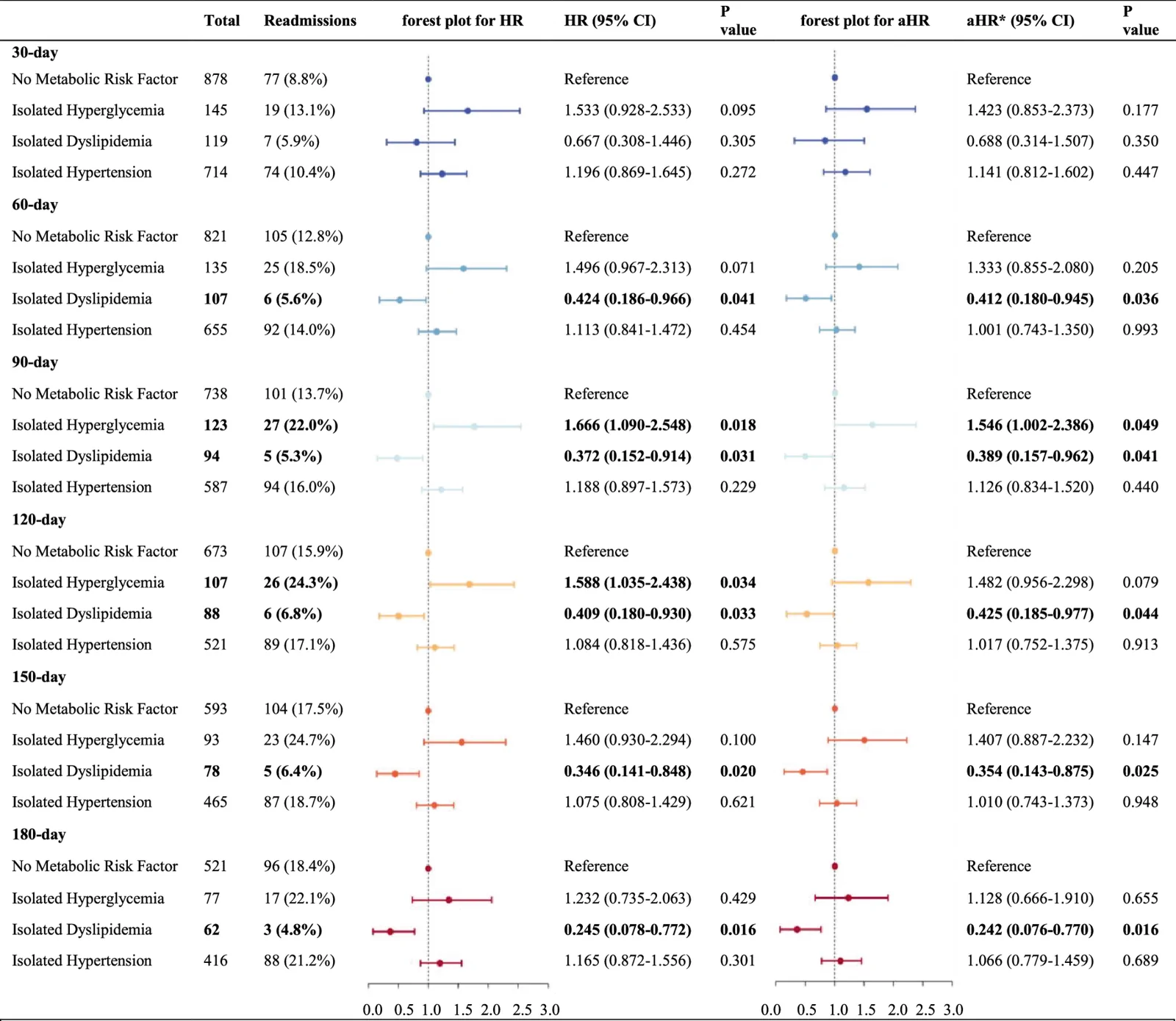
Readmission risk by type of metabolic risk factor across six follow-up periods

*aHR* adjusted hazard ratio; *CI* conﬁdence interval;HR, hazard ratio; *MHNO* metabolically healthy nonobese; *MUNO* metabolicallyunhealthy nonobese; *MHO* metabolically healthy obese; *MUO* metabolicallyunhealthy obese

Adjusted COX regression: adjusted for age, sex,elective admission, primary payer, disposition of patient, length of stay,REHABtransfer,total charges,patient location, obesity,history ofchemotherapy,tobacco,alcohol abuse

When patients were stratified by the number of metabolic risk factors (Table 4), those with two or three metabolic risk factors had higher readmission risks than those with none. Patients with only one metabolic risk factor did not differ significantly from the reference group. Overall, readmission risk increased as the number of concurrent metabolic risk factors increased.

**Table 4:**
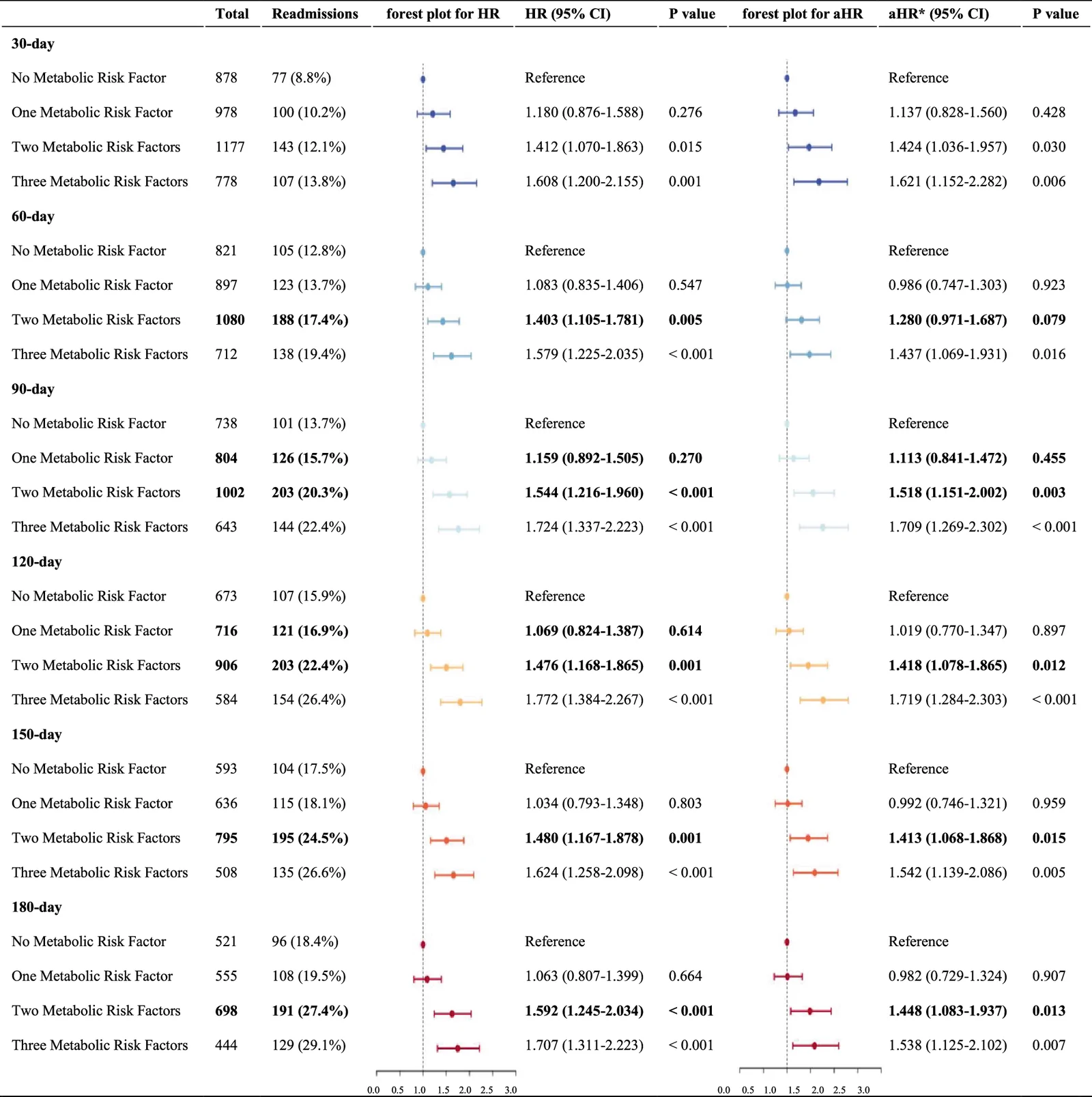
Readmission risk by number of metabolic risk factors across six follow-up periods

*Adjusted COX regression: adjusted for age, sex,elective admission, primary payer, disposition of patient, length of stay, REHABtransfer, total charges, patient location, obesity, history ofchemotherapy, tobacco,alcohol abuse

Subgroup analysis by obesity severity (Table 5) showed that patients with class II obesity had a lower readmission risk than patients with normal weight in the 30-, 60-, 90-, 120-, and 150-day follow-up cohorts. In the 30-day cohort, a reduced readmission risk was also observed among patients with class I obesity. After adjustment, this protective association remained statistically significant only for class II obesity at 30 days.

**Table 5:**
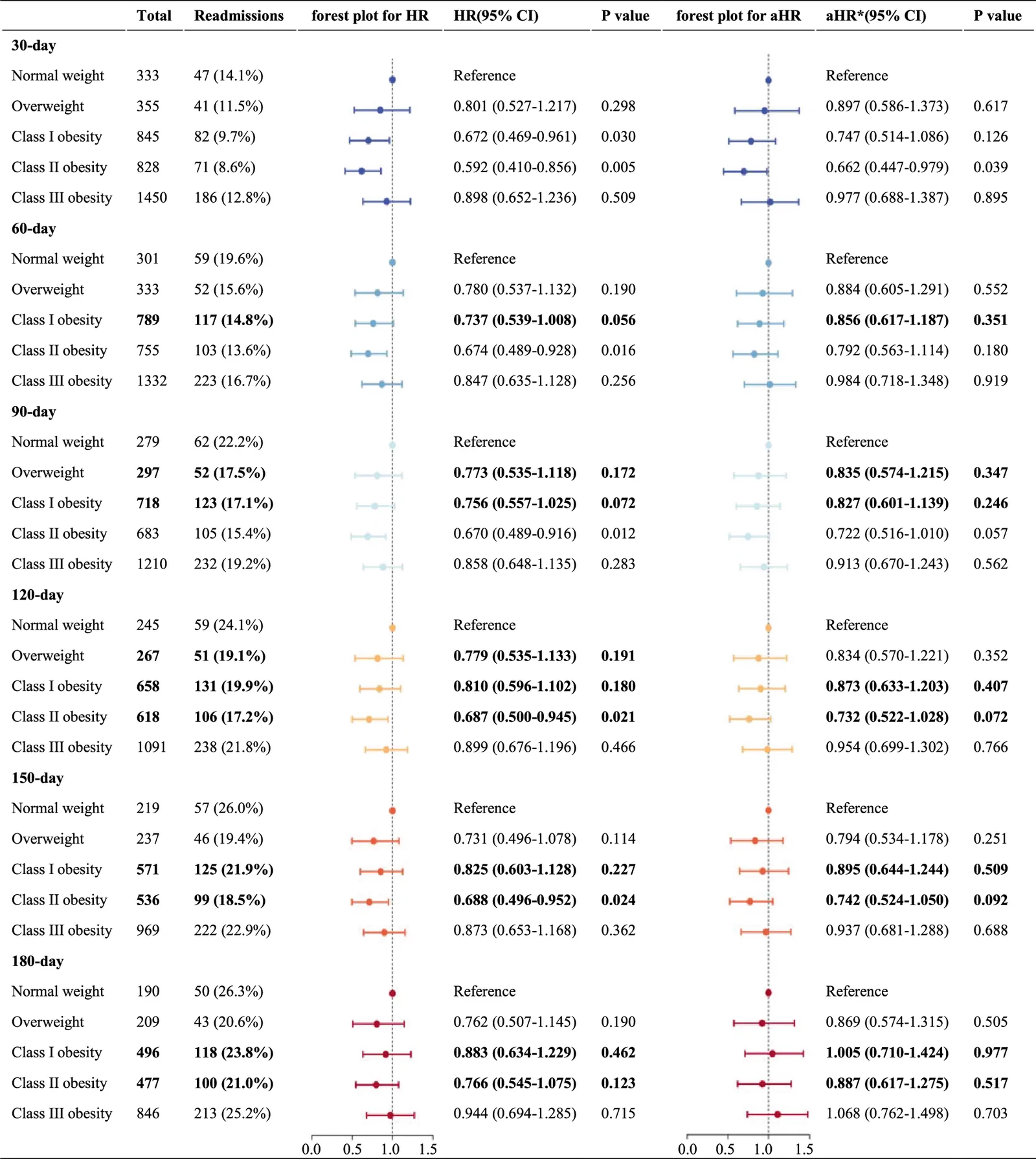
Readmission risk by BMI category across six follow-up periods

*Adjusted COX regression: adjusted for age, sex,elective admission, primary payer, disposition of patient, length ofstay,REHABtransfer,total charges,patient location,metabolic risk factor factorand numbers,history of chemotherapy,tobacco, alcohol abuse

Further stratified analyses of the 120-, 150-, and 180-day follow-up cohorts provided additional findings. In age-stratified analyses, the MUO group had a higher readmission risk than the MHNO group, and this association was observed only among patients younger than 65 years at 150 and 180 days (Table 6). In sex-stratified analyses, MUO was associated with a higher readmission risk than MHNO among female patients (Table 6). In addition, in the 120-day cohort, MUNO was associated with a higher readmission risk than MHNO among female patients.

**Table 6:** Readmission risk stratified by age and sex across three follow-up periods

Variables		population	aHR (95% CI)	*P* value	population	aHR (95% CI)	*P* value	population	aHR (95% CI)	*P* value
	Age groups	120-day			150-day			180-day		
**MHNO**	**<65 years**	76	Reference		71	Reference	65		Reference	
**MUNO**		15	1.656 (0.537–2.099)	0.380	11	2.249 (0.628–8.054)	0.213	8	2.223 (0.480-10.301)	0.307
**MHO**		897	0.997 (0.562–1.769)	0.991	794	1.031 (0.568–1.871)	0.920	690	1.156 (0.608–2.201)	0.658
**MUO**		566	1.746 (0.991–3.075)	0.054	**504**	**1.820 (1.012–3.273)**	**0.045**	**445**	**2.354 (1.251–4.429)**	**0.008**
**MHNO**	≥ **65 years**	74	Reference		64	Reference		54	Reference	
**MUNO**		77	1.606 (0.832–3.097)	0.158	70	1.778 (0.910–3.474)	0.092	60	1.771 (0.878–3.570)	0.110
**MHO**		318	1.287 (0.693–2.391)	0.424	277	1.397 (0.741–2.635)	0.302	246	1.493 (0.774–2.878)	0.232
**MUO**		822	1.468 (0.818–2.634)	0.198	709	1.406 (0.769–2.572)	0.268	622	1.371 (0.732–2.565)	0.324
	**Sex**	**120-day**			**150-day**			**180-day**		
**MHNO**	**Female**	119	Reference		63	Reference		54	Reference	
**MUNO**		**68**	**1.973 (1.073–3.629)**	**0.029**	33	2.025 (0.854–4.799)	0.109	26	2.316 (0.930–5.767)	0.071
**MHO**		936	1.135 (0.702–1.835)	0.607	747	1.487 (0.777–2.845)	0.231	652	1.677 (0.847–3.319)	0.138
**MUO**		**1067**	**1.800 (1.135–2.856)**	**0.012**	**667**	**1.986 (1.052–3.750)**	**0.034**	**584**	**2.296 (1.175–4.490)**	**0.015**
**MHNO**	**Male**	79	Reference		72	Reference		65	Reference	
**MUNO**		56	1.452 (0.718–2.937)	0.299	48	1.893 (0.930–3.856)	0.079	42	1.775 (0.824–3.824)	0.143
**MHO**		368	0.742 (0.408–1.350)	0.329	324	0.794 (0.429–1.469)	0.463	284	0.834 (0.435–1.601)	0.586
**MUO**		626	1.068 (0.613–1.862)	0.817	546	1.146 (0.648–2.030)	0.639	483	1.300 (0.712–2.372)	0.393

These subgroup findings suggest that the association between metabolically unhealthy phenotypes and readmission may differ by age and sex. However, multiple statistical tests were performed across six follow-up intervals and several stratified subgroups, which may increase the probability of false-positive findings. The significant associations observed at 120, 150, and 180 days were consistently replicated across all three mid- to long-term follow-up intervals and were validated in sensitivity analyses using an alternative BMI cutoff. These findings reduce the likelihood that the observed associations were attributable solely to chance from multiple comparisons. Significant findings limited to a single time point or subgroup were therefore interpreted as exploratory signals rather than definitive conclusions. Because this analysis did not include a non-ITP comparator cohort, it remains uncertain whether these associations are specific to ITP or reflect a broader effect of metabolic comorbidity burden on hospital readmission risk.

### Hospitalization burden

Across the 30-, 60-, 90-, 120-, 150-, and 180-day follow-up cohorts, readmission occurred in 427 (11.2%), 554 (15.8%), 574 (18.0%), 585 (20.3%), 549 (21.7%), and 524 (23.6%) patients, respectively. Hospitalization burden stratified by phenotype is summarized in Table 7. Compared with the MHNO and MUNO groups, overweight or obese patients had a shorter mean LOS and a lower proportion of hospitalizations lasting more than 7 days. Compared with the MUO group, the MHO group had a lower proportion of LOS exceeding 7 days, whereas the MUNO group had a higher proportion of LOS exceeding 7 days.

**Table 7:** LOS (days) and Total Charges ($) among different metabolic overweight/obesity phenotypes in six follow-up periods

Variables	MHNO	MUNO	MHO	MUO	Total	*P* value
**30-day**						
LOS(days)(mean(SD))	10.2(12.6)^a^	**12.1(13.6)** ^**a**^	6.5(10.8)^b^	6.8(7.6)^b^	7.0(9.7)	**< 0.001**
LOS > 7 days	87(43.3%)^a^	**68(51.5%)** ^**a**^	358(21.6%)^b^	509(27.9%)^c^	1022(26.8%)	**< 0.001**
Total Charges($)(mean(SD))	**152854.6 (297837.4)** ^**a**^	135667.8 (179071.3)^a, b^	92472.2 (149817.2)^c^	95292.0 (125285.4)^b, c^	98501.9 (152426.1)	**0.002**
Total Charges>$50,000	124(61.7%)^a^	**82(62.1%)** ^**a**^	795(48.0%)^b^	968(53.1%)^a^	1969(51.7%)	**< 0.001**
**60-day**						
LOS(days)(mean(SD))	9.5(10.0)^a^	**11.8(13.2)** ^**a**^	6.4(8.9)^b^	6.8(7.6)^b^	6.9(8.6)	**< 0.001**
LOS > 7 days	79(42.9%)^a^	**60(51.3%)** ^**a**^	331(21.6%)^b^	462(27.6%)^c^	932(26.6%)	**< 0.001**
Total Charges($)(mean(SD))	143562.9 (270210.5)^a^	135815.8 (177170.7)^a^	92244.0 (151815.6)^a^	94289.3 (120489.4)^a^	97362.7 (148383.1)	**0.006**
Total Charges>$50,000	112(60.9%)^a^	**72(61.5%)** ^**a**^	734(47.8%)^b^	880(52.5%)^a^	1798(51.2%)	**< 0.001**
**90-day**						
LOS(days)(mean(SD))	9.6(10.2)^a^	**11.9(13.8)** ^**a**^	6.3(8.7)^b^	6.8(7.6)^b^	6.9(8.6)	**< 0.001**
LOS > 7 days	76(43.7%)^a^	**52(49.5%)** ^**a**^	293(21.4%)^b^	427(27.7%)^c^	848(26.6%)	**< 0.001**
Total Charges($)(mean(SD))	145949.0 (276842.9)^a^	136742.9 (184094.0)^a^	90436.5 (150227.3)^a^	92969.0 (117798.5)^a^	96216.7 (147861.0)	**0.006**
Total Charges>$50,000	**106(60.9%)** ^**a**^	62(59.0%)^a, b^	651(47.6%)^b^	802(52.1%)^a, b^	1621(50.9%)	**< 0.001**
**120-day**						
LOS(days)(mean(SD))	9.5(10.4)^a^	**12.3(14.4)** ^**a**^	6.2(8.3)^b^	6.8(7.7)^b^	6.9(8.5)	**< 0.001**
LOS > 7 days	67(43.8%)^a^	**47(51.1%)** ^**a**^	264(21.4%)^b^	390(27.9%)^c^	768(26.7%)	**< 0.001**
Total Charges($)(mean(SD))	134679.1 (188100.2)^a^	**138610.2 (186913.3)** ^**a, b**^	89429.6 (135005.4)^b^	94309.5 (120605.1)^a, b^	95775.5 (134211.0)	**0.004**
Total Charges>$50,000	91(59.5%)^a^	**56(60.9%)** ^**a, b**^	585(47.3%)^b^	732(52.4%)^a, b^	1464(50.9%)	**< 0.001**
**150-day**						
LOS(days)(mean(SD))	9.6(10.7)^a^	**11.8(11.8)** ^**a**^	6.1(7.6)^b^	6.8(7.6)^b^	6.8(8.1)	**< 0.001**
LOS > 7 days	60(43.5%)^a^	**42(51.9%)** ^**a**^	233(21.4%)^b^	346(28.3%)^c^	681(26.9%)	**< 0.001**
Total Charges($)(mean(SD))	135229.2 (193902.8)^a^	146773.3 (196358.6)^a^	89920.1 (136636.3)^a^	93521.0 (119741.5)^a^	95946.2 (135632.9)	**0.005**
Total Charges>$50,000	83(60.1%)^a^	**51(63.0%)** ^**a**^	516(47.3%)^b^	636(52.0%)^a, b^	1286(50.8%)	**< 0.001**
**180-day**						
LOS(days)(mean(SD))	10.0(11.2)^a^	**11.8(11.9)** ^**a**^	6.2(7.6)^b^	6.7(7.2)^b^	6.8(7.9)	**< 0.001**
LOS > 7 days	57(46.7%)^a^	**34(50.0%)** ^**a**^	209(21.9%)^b^	301(28.0%)^c^	601(27.1%)	**< 0.001**
Total Charges($)(mean(SD))	142196.7 (203322.1)^a^	**149096.0 (206842.4)** ^**a, b**^	90870.8 (139085.9)^b^	91030.7 (113195.4)^b^	95556.5 (135252.5)	**0.006**
Total Charges > $50,000	75(61.5%)^a^	**42(61.8%)** ^**a, b**^	455(47.7%)^b^	554(51.6%)^a, b^	1126(50.8%)	**0.005**

In the 30- and 60-day cohorts, the MHO group had the lowest proportion of patients with total hospital charges exceeding $ 50,000. In the 90-, 120-, 150-, and 180-day cohorts, the proportion of patients with total hospital charges exceeding $ 50,000 was lower in the MHO group than in the MHNO group. In the 30- and 180-day cohorts, the MHO and MUO groups had lower mean total hospital charges than the MHNO group. In the 60-day cohort, mean total hospital charges were lower in the MHO group than in the MHNO group. However, in the 30-day cohort, the MUNO group had higher mean total hospital charges than the MHO group. This pattern was observed in the present ITP cohort, but whether it is specific to ITP remains uncertain.

These findings suggest that metabolically unhealthy status is associated with greater hospitalization burden. An inverse association was also observed between overweight/obesity and hospitalization burden, with overweight/obesity appearing to correlate with shorter LOS and lower medical charges. Several explanations may account for these findings. Metabolic disorders may be associated with less favorable disease trajectories, poorer treatment responses, and prolonged hospitalization, thereby increasing medical costs. In contrast, higher BMI among obese patients may reflect greater physiological reserve, better treatment tolerance, and less severe disease-related weight loss.

### Hospitalization outcomes and mortality during readmission

Among patients discharged after the index hospitalization and followed for 30, 60, 90, 120, 150, and 180 days, readmission occurred in 427 (11.2%), 554 (15.8%), 574 (18.0%), 585 (20.3%), 549 (21.7%), and 524 (23.6%) cases, respectively.

During readmission, the MUNO group had a higher proportion of patients with LOS exceeding 3 days than the MHNO group at 90 days (75.9% vs. 69.7%, *P* = 0.038), 120 days (78.6% vs. 74.2%, *P* = 0.022), 150 days (78.6% vs. 72.4%, *P* = 0.019), and 180 days (79.2% vs. 73.1%, *P* = 0.020) (Table 8). However, because of the limited sample size, no significant differences in LOS, total charges, or readmission mortality were detected among the four phenotypes.

**Table 8:** Hospitalization outcomes and mortality during readmission across six follow-up periods

Variables	MHNO	MUNO	MHO	MUO	Total	*P* value
**30-day**						
Total Charges($)(mean(SD))	92817.9 (100640.7)	81540.68 (79852.6)	94297.9 (132993.3)	97160.8 (163745.2)	95082.6 (146472.1)	0.970
LOS(days)(mean(SD))	7.60 (6.79)	6.14 (4.14)	6.86 (7.54)	6.93 (8.20)	6.90 (7.71)	0.934
LOS > 3 days	16 (64.0%)	14 (63.6%)	89 (58.6%)	133 (58.3%)	252 (59.0%)	0.917
Died	1 (4.0%)	2 (9.1%)	10 (6.6%)	13 (5.7%)	26 (6.1%)	0.887
**60-day**						
Total Charges($)(mean(SD))	95532.0 (92577.5)	81779.4 (81625.6)	91881.2 (134382.4)	92416.0 (154841.4)	91862.6 (141681.3)	0.983
LOS(days)(mean(SD))	7.90 (7.21)	6.61 (4.69)	6.45 (7.43)	6.76 (7.80)	6.71 (7.50)	0.793
LOS > 3 days	21 (67.7%)	20 (71.4%)	108 (54.8%)	170 (57.0%)	319 (57.6%)	0.245
Died	1 (3.2%)	2 (7.1%)	10 (5.1%)	19 (6.4%)	32 (5.8%)	0.825
**90-day**						
Total Charges($)(mean(SD))	130027.2 (177484.3)	79326.6 (79442.6)	93394.5 (146875.4)	85331.7 (136532.0)	90323.0 (140571.5)	0.348
LOS(days)(mean(SD))	8.70 (8.28)	6.66 (4.54)	6.41 (7.67)	6.57 (7.70)	6.64 (7.60)	0.453
LOS > 3 days	**23 (69.7%)** ^**a**^	**22 (75.9%)** ^**a**^	**101 (52.1%)** ^**a**^	**176 (55.3%)** ^**a**^	**322 (56.1%)**	**0.038**
Died	1 (3.0%)	3 (10.3%)	11 (5.7%)	19 (6.0%)	34 (5.9%)	0.691
**120-day**						
Total Charges($)(mean(SD))	135816.5 (182514.6)	78731.0 (82590.0)	97761.7 (148045.6)	85294.0 (133683.1)	91855.6 (139880.4)	0.223
LOS(days)(mean(SD))	9.45 (9.86)	7.89 (8.80)	6.83 (7.77)	6.53 (7.56)	6.85 (7.84)	0.216
LOS > 3 days	**23 (74.2%)** ^**a**^	**22 (78.6%)** ^**a**^	**107 (54.3%)** ^**a**^	**185 (56.2%)** ^**a**^	**337 (57.6%)**	**0.022**
Died	1 (3.2%)	3 (10.7%)	10 (5.1%)	18 (5.5%)	32 (5.5%)	0.661
**150-day**						
Total Charges($)(mean(SD))	135126.3 (189919.0)	66128.9 (58592.9)	93417.1 (139101.1)	85475.5 (138730.5)	89860.0 (139316.0)	0.231
LOS(days)(mean(SD))	9.66 (10.21)	6.50 (4.06)	6.73 (7.88)	6.23 (7.43)	6.60 (7.62)	0.358
LOS > 3 days	**21 (72.4%)** ^**a**^	**22 (78.6%)** ^**a**^	**101 (53.2%)** ^**a**^	**164 (54.3%)** ^**a**^	**308 (56.1%)**	**0.019**
Died	1 (3.4%)	3 (10.7%)	10 (5.3%)	16 (5.3%)	30 (5.5%)	0.688
**180-day**						
Total Charges($)(mean(SD))	97695.4 (97184.1)	66999.3 (58993.9)	92875.5 (141231.1)	87754.7 (140634.0)	89036.8 (136171.7)	0.826
LOS(days)(mean(SD))	7.77 (6.43)	6.71 (4.25)	6.64 (7.83)	6.40 (7.63)	6.56 (7.51)	0.839
LOS > 3 days	**19 (73.1%)** ^**a**^	**19 (79.2%)** ^**a**^	**92 (51.7%)** ^**a**^	**161 (54.4%)** ^**a**^	**291 (55.5%)**	**0.020**
Died	1 (3.8%)	3 (12.5%)	10 (5.6%)	15 (5.1%)	29 (5.5%)	0.581

### Distribution of discharge diagnoses among readmitted patients

The distribution of principal discharge diagnoses during readmission was assessed for diagnoses that accounted for at least 1% of readmission events or ranked among the top 10 diagnoses among patients with ITP at each follow-up interval from 30 to 180 days (Table 9). ITP (D69.3) was the most common principal diagnosis at all follow-up time points, accounting for 19.8%-20.3% of readmissions. We further examined the distribution of ITP among the second to tenth ranked discharge diagnoses and calculated the cumulative number of ITP cases among the 10 most common diagnoses (Table 10). Overall, patients with ITP listed among the top 10 discharge diagnoses accounted for 65.7%-66.4% of all readmission cases.

**Table 9:** Distribution of principal discharge diagnoses among readmitted patients

1st diagnosis (Top10 or ratio ≥ 1%)	30-day	60-day	90-day	120-day	150-day	180-day
ITP	757 (19.9%)	711 (20.3%)	630 (19.8%)	572 (19.9%)	515 (20.3%)	449 (20.2%)
Sepsis	212 (5.6%)	193 (5.5%)	180 (5.6%)	165 (5.7%)	149 (5.9%)	127 (5.7%)
Pneumonia	63(1.7%)	58 (1.7%)	53 (1.7%)	46 (1.6%)	43 (1.7%)	38 (1.7%)
Acute kidney failure	61 (1.6%)	58 (1.7%)	50 (1.6%)	46 (1.6%)	44 (1.7%)	41 (1.8%)
Hypertensive heart and chronic kidney disease with heart failure and stage 1–4 or unspecified chronic kidney disease	60 (1.6%)	54 (1.5%)	53 (1.7%)	52 (1.8%)	46 (1.8%)	39 (1.8%)
Hypertensive heart disease with heart failure	56 (1.5%)	47 (1.6%)	44 (1.4%)	40 (1.4%)	36 (1.4%	34 (1.5%)
NSTEMI	56 (1.5%)	53 (1.5%)	47 (1.5%)	45 (1.6%)	38 (1.5%))	33 (1.5%)
Morbid (severe) obesity due to excess calories	49 (1.3%)	44 (1.6%)	39 (1.2%)	37 (1.3%)	33 (1.3%)	26 (1.5%)
Other diseases of the blood and blood-forming organs and certain immune disorders complicating childbirth	43 (1.1%)	40 (1.1%)	38 (1.2%)	35 (1.2%)	31 (1.2%)	25 (1.1%)
Unilateral primary osteoarthritis, right knee	41 (1.1%)	35 (1.0%)	30 (0.9%)	27 (0.9%)	/	/
Acute respiratory failure with hypoxia	/	/	/	/	25 (1.0%)	22 (1.0%)

**Table 10:** Distribution of ITP across the first to tenth discharge diagnosis positions

Diagnostic position with ITP	30-day	60-day	90-day	120-day	150-day	180-day
**1st**	757 (19.9%)	711 (20.3%)	630 (19.8%)	572 (19.9%)	515 (20.3%)	449 (20.2%)
**2nd**	730 (19.2%)	674 (19.2%)	619 (19.4%)	564 (19.6%)	489 (19.3%)	433 (19.5%)
**3rd**	14 (0.4%)	12 (0.3%)	12 (0.4%)	11 (0.4%)	11 (0.4%)	9 (0.4%)
**4th**	78 (2.0%)	69 (2.0%)	61 (1.9%)	54 (1.9%)	43 (1.7%)	36 (1.6%)
**5th**	49 (1.3%)	46 (1.3%)	41 (1.3%)	36 (1.3%)	29 (1.1%)	25 (1.1%)
**6th**	109 (2.9%)	97 (2.8%)	84 (2.6%)	80 (2.8%)	70 (2.8%)	57 (2.6%)
**7th**	387 (10.2%)	358 (10.2%)	335 (10.5%)	301 (10.5%)	261 (10.3%)	224 (10.1%)
**8th**	85 (2.2%)	79 (2.3%)	71 (2.2%)	66 (2.3%)	58 (2.3%)	50 (2.3%)
**9th**	218 (5.7%)	201 (5.7%)	186 (5.8%)	160 (5.6%)	141 (5.6%)	119 (5.4%)
**10th**	83 (2.2%)	81 (2.3%)	75 (2.4%)	68 (2.4%)	61 (2.4%)	55 (2.5%)
**Total**	2510 (65.9%)	2328 (66.3%)	2114 (66.3%)	1912 (66.4%)	1678 (66.3%)	1457 (65.7%)

Among principal discharge diagnoses during readmission, infectious diseases were the second most common category after ITP. Sepsis (A41.9) and pneumonia (J18.9) together accounted for 7.2%-7.6% of readmissions. Cardiovascular and metabolic diseases were also common, including hypertensive heart and renal disease (I13.0), non-ST-elevation myocardial infarction (I21.4), and hypertensive heart disease with heart failure (I11.0), which together accounted for 4.5%-4.8%. Acute kidney failure (N17.9) accounted for 1.6%-1.8% of readmissions. Obesity-related diseases, hematological complications of pregnancy, osteoarticular diseases, and acute respiratory failure were also included among the 10 most common diagnoses. The relative distribution of these diagnoses remained stable throughout the follow-up period.

## Discussion

Using data from the NRD, this study evaluated the association between metabolic overweight/obesity phenotypes and readmission risk among patients with ITP. In this observational cohort, metabolic abnormalities, rather than overweight/obesity itself, were associated with increased mid- to long-term readmission risk. Because a non-ITP inpatient comparator group was not included, these associations may reflect the broader effect of metabolic comorbidity on readmission rather than a pattern specific to ITP. Patients with metabolically unhealthy phenotypes had a significantly higher risk of readmission during mid- to long-term follow-up, whereas metabolically healthy obese patients did not show an increased risk at any follow-up interval. During the short-term follow-up periods of 30 to 90 days, readmission risk did not differ significantly across metabolic overweight/obesity phenotypes. However, compared with metabolically healthy non-obese patients, those with metabolically unhealthy phenotypes had a higher risk of readmission at 120, 150, and 180 days. Readmission risk also increased progressively with the number of concurrent metabolic risk factors.

Patients with the MHO phenotype did not have an increased readmission risk at any follow-up interval. This finding is consistent with the “metabolically healthy obesity” phenotype described in recent studies of several chronic diseases, suggesting that obesity without overt metabolic dysfunction does not necessarily lead to poorer clinical outcomes [14]. Previous studies have suggested that, in certain chronic conditions, a higher body mass index may provide advantages through greater energy and protein reserves and better tolerance of physiological stress. In patients with ITP, these factors may improve tolerance to repeated treatments and reduce treatment-related complications, thereby partially attenuating the risk of disease-related hospitalization [15].

In contrast, metabolically unhealthy phenotypes (MUNO and MUO) are characterized by a higher prevalence of cardiovascular comorbidities than metabolically healthy phenotypes, which may partly explain the observed increase in readmission risk [16–18]. Analysis of discharge diagnoses during readmission showed that ITP relapse was the leading cause of post-discharge readmission. Among non-ITP causes, infectious diseases were the second most common reason for readmission, followed by cardiovascular and metabolic disorders and acute renal failure, all of which ranked among the 10 most common readmission diagnoses. These findings indicate that mid- and long-term readmissions in patients with ITP are not solely attributable to disease recurrence. Comorbid infections and cardiovascular and metabolic conditions also contribute substantially to the readmission burden. From the perspective of readmission diagnosis, metabolic comorbidities appear to be closely associated with higher readmission rates in patients with ITP. These findings are consistent with long-term cohort studies showing that metabolically unhealthy individuals have an increased cardiovascular risk across body mass index categories and that transition from a metabolically healthy to a metabolically unhealthy phenotype is associated with a gradual increase in cardiovascular risk [19]. Compared with patients with MHO, those with MUO typically have more pronounced insulin resistance, unfavorable fat distribution, higher inflammatory marker levels, and adipose tissue dysfunction, all of which are closely related to atherosclerosis and cardiovascular disease [20, 21]. Metabolic disorders may also influence ITP severity, treatment response, and the overall disease course. In addition, patients with metabolically unhealthy profiles may be less likely to receive intensive or optimal management, which could further contribute to adverse clinical outcomes [22, 23].

The inverse association observed in the isolated dyslipidemia subgroup should be interpreted cautiously. This finding may reflect residual confounding, including unmeasured statin use, and may also indicate a lower overall metabolic burden among patients without concomitant hypertension or hyperglycemia. Because this subgroup analysis was exploratory and the association was not consistently significant across all follow-up intervals, no definitive clinical conclusion should be drawn from this result.

An increasing body of evidence indicates that the onset and progression of ITP are not attributable solely to immune dysregulation but are closely related to interactions between immune regulation and metabolic homeostasis [24, 25]. Compared with metabolically unhealthy individuals, patients with metabolically healthy obesity generally have lower levels of adipose tissue–derived inflammatory mediators and pro-inflammatory adipokines. These relatively lower inflammatory signals may be insufficient to substantially amplify autoimmune responses and may therefore have a limited effect on the clinical course and relapse risk of ITP [26, 27]. Obesity is characterized by chronic low-grade inflammation, which is triggered by adipocyte hypertrophy and increased secretion of pro-inflammatory cytokines, including interleukin-6, interleukin-1, interleukin-8, and tumor necrosis factor-α, as well as adipokines such as leptin, adiponectin, resistin, and visfatin secreted by adipose tissue [28, 29]. Elevated interleukin-6 levels stimulate hepatic production of C-reactive protein, and higher C-reactive protein concentrations have been associated with lower platelet counts [30]. Leptin further promotes the release of inflammatory cytokines, which in turn upregulate leptin expression, forming a positive feedback loop that maintains chronic inflammation [31]. In addition, adiponectin can activate the alternative complement pathway through cleavage of complement factor B. Complement activation may contribute to platelet destruction in ITP, even in the absence of detectable autoantibodies [32]. Maintenance of a metabolically healthy state may also help preserve responsiveness to glucocorticoids, immunosuppressive agents, or thrombopoietin receptor agonists, thereby reducing the likelihood of treatment failure or disease relapse that could ultimately result in hospital readmission [33].

In addition to these conventional inflammatory mediators, emerging mechanistic evidence links metabolically unhealthy phenotypes to ITP pathophysiology through chronic low-grade inflammation and inflammasome-mediated pyroptosis. Metabolically unhealthy status, characterized by insulin resistance and lipid dysregulation, creates a persistent inflammatory milieu that serves as an important priming signal for activation of the NLRP3 inflammasome, a central sensor linking metabolic stress with autoimmune inflammation [34]. Aberrant assembly of the NLRP3 inflammasome subsequently induces caspase-1-dependent pyroptosis, a pro-inflammatory form of programmed cell death that promotes the maturation and release of IL-1β and IL-18. This process may further amplify systemic inflammation and autoimmune platelet destruction [35]. Accumulating clinical evidence has shown enhanced NLRP3 inflammasome activity and elevated downstream inflammatory cytokines in patients with active ITP, with close correlations with disease severity and thrombocytopenia [36]. Platelets and immune cells from patients with ITP can undergo inflammasome-mediated pyroptosis, which may directly accelerate platelet depletion and perpetuate disease relapse [37]. Additionally, lipid abnormalities induced by metabolic disturbance may further potentiate inflammasome activation, creating a self-reinforcing cycle that exacerbates immune dysregulation and unfavorable clinical outcomes in patients with ITP and metabolic comorbidities [38]. Although these mechanisms may biologically explain readmissions due to ITP relapse, the absence of a non-ITP control group in this study prevents us from determining whether the elevated risk is driven by immune-metabolic disturbances specific to ITP or by the general readmission risk associated with common metabolic diseases.

Notably, the association between metabolically unhealthy status and readmission emerged at 120 days and persisted at 150 and 180 days, whereas no significant association was observed at 30, 60, or 90 days. This temporal pattern suggests that metabolic abnormalities may exert delayed or cumulative effects on outcomes in ITP. However, without a non-ITP control group, this pattern may also reflect a general feature of hospitalized patients with metabolic comorbidities rather than a characteristic specific to ITP. In clinical practice, short-term readmissions in ITP are more often driven by acute bleeding events, inadequate initial treatment response, or concomitant infections. By contrast, readmissions during mid- to long-term follow-up are more likely to be related to disease relapse, failure during treatment tapering, or suboptimal management during the maintenance phase. In this context, Danese et al. [39] reported that both the risk and severity of ITP-related hospitalizations increased significantly with age. However, across all age groups, the relative mortality risk of patients with ITP compared with hospitalized patients without ITP remained stable rather than being limited to acute disease episodes. This observation is consistent with our finding that differences in readmission risk were not prominent in the early post-discharge period but became evident during longer-term follow-up. Together, these findings support the concept that the burden of ITP is mediated mainly through chronic and cumulative mechanisms rather than isolated acute events.

Traditional management of ITP has primarily focused on platelet count and bleeding risk as the main clinical endpoints. However, our findings suggest that, in real-world populations, metabolic abnormalities may identify patients at increased risk of mid- to long-term readmission, with this risk becoming more apparent during later follow-up. This observation indicates that management strategies focused solely on short-term hemostasis or platelet elevation may be insufficient to reduce longer-term healthcare utilization [40, 41]. In addition to routine evaluation of platelet counts, bleeding phenotype, and treatment response, discharge planning and post-discharge follow-up for patients with ITP should include systematic assessment and longitudinal management of metabolic parameters, including blood glucose, blood pressure, and lipid profiles. Patients with concomitant metabolic abnormalities may benefit from closer surveillance, which could allow earlier recognition of clinical trajectories associated with recurrent hospitalization. ITP should not be regarded solely as a bleeding disorder. Accumulating evidence indicates that adult patients with ITP also have a higher burden of thrombotic events and cardiovascular risk factors [42]. Therefore, identification and targeted management of metabolic risk factors may have broader clinical relevance for reducing readmission risk and improving long-term outcomes. Because metabolic risk factors are potentially modifiable through lifestyle intervention and pharmacologic treatment, optimizing metabolic health in patients with ITP may represent a feasible strategy to attenuate disease progression and reduce the likelihood of readmission [9, 43].

Several limitations should be acknowledged. First, this study was based on administrative claims data rather than a prospective clinical registry, which inevitably introduced residual confounding and information bias. Although our analyses adjusted for demographic characteristics, admission-related variables, behavioral factors, and chronic comorbidities, the NRD does not provide key ITP-specific clinical information. Unavailable variables included baseline platelet counts, disease phase, bleeding severity grade, detailed treatment regimens, treatment response, follow-up adherence, and routine laboratory findings. These factors are independent predictors of ITP relapse and readmission. If they were unevenly distributed across metabolic overweight/obesity phenotypes, the true magnitude of the observed associations may have been distorted. The lack of prescription data for statins and the inability to capture unmeasured organ injury also limited interpretation of the inverse association observed in the isolated dyslipidemia subgroup. Second, metabolic health status was defined using diagnostic codes rather than continuous laboratory measurements or repeated longitudinal metabolic assessments. This approach may have underestimated subclinical metabolic abnormalities and led to phenotype misclassification. Third, consistent with similar real-world database studies examining metabolic complications and readmission risk in hematological malignancies [44, 45], BMI ≥ 25 kg/m² was used as the cutoff for overweight/obesity. This threshold facilitates comparison with published evidence and supports cross-study consistency. Sensitivity analyses using the conventional WHO obesity threshold of BMI ≥ 30 kg/m² confirmed the robustness of the main findings. Nevertheless, BMI cannot distinguish visceral adiposity from subcutaneous fat, nor can it capture muscle mass or other body composition features that may be closely related to immunometabolic regulation. This represents an inherent limitation of administrative database studies. Fourth, multiple statistical comparisons were performed across follow-up intervals, phenotypic subgroups, and stratified analyses. Although Bonferroni correction was applied, residual inflation of type I error cannot be fully excluded. Associations observed only in a single subgroup or at a single time point should therefore be interpreted cautiously and not overextended. In contrast, the main findings that were reproduced across multiple follow-up intervals and supported by sensitivity analyses are more robust. Finally, because a non-ITP comparator cohort was not available, this study could not determine whether the association between metabolically unhealthy phenotypes and readmission was specific to ITP or reflected a broader effect of metabolic comorbidity burden on hospital readmission. In addition, unavailable socioeconomic variables and loss of interstate readmissions in the NRD further limited causal interpretation. Given the retrospective observational design and unavoidable residual confounding, the present findings should be interpreted as associations rather than evidence of causality.

Despite these limitations, this study provides population-based evidence from a nationally representative cohort with extended follow-up and patient-level readmission tracking. These features allowed us to characterize heterogeneity in mid- to long-term readmission risk among patients with ITP according to metabolic overweight/obesity phenotype. Future studies should validate these findings in prospective cohorts that incorporate dynamic measures of ITP activity, treatment exposure, treatment response, and metabolic status. Further research is also needed to determine whether active optimization of metabolic health can reduce readmission rates in patients with ITP and thereby support translation of these findings into clinically actionable follow-up strategies.

## Conclusion

In this national retrospective cohort study, metabolic abnormalities, rather than overweight/obesity alone, were independently associated with an increased risk of readmission among patients with ITP after multivariable adjustment. This association was supported by sensitivity analysis using the conventional BMI ≥ 30 kg/m² obesity threshold, and the consistency of the findings across multiple follow-up intervals reduced the likelihood that the results were driven by chance alone. Readmission risk increased in parallel with the number of concurrent metabolic risk factors. However, because no non-ITP control group was available, it remains unclear whether these associations are specific to ITP or reflect a general effect of metabolic comorbidities on hospital readmission. Prospective studies with detailed clinical, laboratory, and treatment data are warranted to reduce residual confounding and to determine whether metabolic management can lower readmission risk in this population.

## Supplementary Information

Below is the link to the electronic supplementary material.

Supplementary Material 1

## Data Availability

The datasets used in this study were obtained from the Healthcare Cost and Utilization Project (HCUP) National Readmissions Database (NRD), sponsored by the Agency for Healthcare Research and Quality (AHRQ). Access to NRD data is available to researchers who sign a Data Use Agreement (DUA) and complete the required HCUP Data Use Agreement Training Course. For more information or to apply for access, please visit the following HCUP websites: https://www.hcup-us.ahrq.gov/db/nation/nrd/nrddbdocumentation.jsp and https://www.hcup-us.ahrq.gov/tech_assist/centdist.jsp .
