# Supplementary material for: Association between metabolic overweight/obesity phenotypes and readmission risk in patients with Immune Thrombocytopenia (ITP): a national retrospective study
Source: Ann Hematol. 2026 Jul 23;105(11):464. doi: 10.1007/s00277-026-07182-y (PMC13680538; doi:10.1007/s00277-026-07182-y)
Supplement: Supplementary file 1 [file 277_2026_7182_MOESM1_ESM.docx]

**Supplementary Text 1**

**Methods**

Patients were reclassified using an alternative BMI cutoff. Patients with BMI <30 kg/m² were categorized as having no obesity, whereas those with BMI ≥30 kg/m² were categorized as having obesity. Based on these BMI categories and metabolic status, four alternative metabolic obesity phenotypes were defined: metabolically healthy without obesity (MHNO), metabolically unhealthy without obesity (MUNO), metabolically healthy with obesity (MHO), and metabolically unhealthy with obesity (MUO). All corresponding diagnoses were identified using ICD-10 codes (Supplementary Table 4).

**Results**

Consistent with the primary analysis based on the BMI ≥25 kg/m² cutoff, the sensitivity analysis using BMI ≥30 kg/m² showed that the core associations between metabolic status and all-cause readmission risk among patients with ITP remained highly stable.

1. Time-dependent readmission risk pattern unchanged

No significant between-group differences in readmission hazard were observed within 30 to 90 days after discharge. Beginning at 120 days after the index hospitalization, both the MUNO and MUO phenotypes were associated with significantly higher adjusted readmission hazards than the MHNO reference group. These associations persisted through the 150-day and 180-day follow-up periods (Supplementary Table 1). In contrast, the MHO phenotype was not associated with increased readmission risk at any follow-up time point, fully replicating the primary findings.

2.Age- and sex-stratified subgroup consistency

Sensitivity analyses stratified by age (<65 vs. ≥65 years) and sex (female vs. male) showed effect modification patterns consistent with those in the main analysis (Supplementary Table 2). The excess readmission risk associated with the MUO phenotype was evident only among patients younger than 65 years and among female patients. No significant increase in risk was detected among older patients or male patients.

3.Hospitalization burden trends replicated

Across all six follow-up intervals, patients without obesity (MHNO and MUNO) had longer mean lengths of stay and higher proportions of hospitalizations exceeding 7 days than patients with obesity (MHO and MUO). The MHO subgroup consistently had lower medical charges than the metabolically unhealthy phenotypes, consistent with the results shown in Table 7 of the primary analysis (Supplementary Table 3).

Changing the obesity cutoff from BMI ≥25 kg/m² to BMI ≥30 kg/m² did not alter the central inference. Metabolic comorbidities, rather than obesity itself, were independently associated with elevated mid- to long-term readmission risk among patients with ITP. The delayed effect of metabolic risk, which became significant only after 120 days, also remained unchanged after reclassification. These findings confirm that the primary results were not driven by the BMI threshold used to define obesity.

Supplementary Table 1. Readmission risk across six follow-up periods by different metabolic overweight/obesity phenotypes.

| **Group** | **Total** | **Readmissions** | | **HR (95% CI)** | **P value** |  | **aHR* (95% CI)** | **P value** | |  |
| --- | --- | --- | --- | --- | --- | --- | --- | --- | --- | --- |
|  | **30-day** |  |  |  |  |  |  | |  | |
| **MHNO** | 381 | 43 (11.3%) |  | Reference | |  | Reference | | | |
| **MUNO** | 307 | 45 (14.7%) |  | 1.305 (0.859-1.982) | 0.212 |  | 1.351 (0.889-2.054) | | 0.159 | |
| **MHO** | 1475 | 134 (9.1%) |  | 0.793 (0.562-1.118) | 0.186 |  | 0.866 (0.613-1.224) | | 0.415 | |
| **MUO** | 1648 | 205 (12.4%) |  | 1.106 (0.796-1.536) | 0.550 |  | 1.187 (0.853-1.653) | | 0.309 | |
|  | **60-day** |  |  |  |  |  |  | |  | |
| **MHNO** | 350 | 54 (15.4%) |  | Reference | |  | Reference | | | |
| **MUNO** | 282 | 56 (19.9%) |  | 1.325 (0.912-1.925) | 0.140 |  | 1.338 (0.911-1.966) | | 0.138 | |
| **MHO** | 1368 | 174 (12.7%) |  | 0.817 (0.602-1.108) | 0.194 |  | 4.159 (0.471-36.718) | | 0.200 | |
| **MUO** | 1510 | 270 (17.9%) |  | 1.183 (0.883-1.584) | 0.260 |  | 5.584 (0.642-48.571) | | 0.119 | |
|  | **90-day** |  |  |  |  |  |  | |  | |
| **MHNO** | 320 | 55 (17.2%) |  | Reference | |  | Reference | | | |
| **MUNO** | 255 | 59 (23.1%) |  | 1.406 (0.973-2.029) | 0.069 |  | 1.437 (0.987-2.094) | | 0.059 | |
| **MHO** | 1222 | 172 (14.1%) |  | 0.811 (0.599-1.099) | 0.177 |  | 0.912 (0.664-1.253) | | 0.571 | |
| **MUO** | 1390 | 288 (20.7%) |  | 1.243 (0.931-1.658) | 0.140 |  | 1.334 (0.993-1.793) | | 0.056 | |
|  | **120-day** |  |  |  |  |  |  | |  | |
| **MHNO** | 280 | 52 (18.6%) |  | Reference | |  | Reference | | | |
| **MUNO** | 231 | 58 (25.1%) |  | 1.432 (0.985-2.082) | 0.060 |  | **1.461 (1.005-2.124)** | | **0.047** | |
| **MHO** | 1109 | 176 (15.9%) |  | 0.846 (0.621-1.153) | 0.290 |  | 0.917 (0.672-1.251) | | 0.584 | |
| **MUO** | 1259 | 299 (23.7%) |  | 1.332 (0.992-1.788) | 0.057 |  | **1.406 (1.047-1.888)** | | **0.024** | |
|  | **150-day** |  |  |  |  |  |  | |  | |
| **MHNO** | 254 | 51 (20.1%) |  | Reference | |  | Reference | | | |
| **MUNO** | 201 | 52 (25.9%) |  | 1.369 (0.930-2.014) | 0.111 |  | 1.405 (0.946-2.087) | | 0.092 | |
| **MHO** | 975 | 168 (17.2%) |  | 0.847 (0.619-1.159) | 0.300 |  | 0.970 (0.700-1.345) | | 0.857 | |
| **MUO** | 1102 | 278 (25.2%) |  | 1.318 (0.978-1.777) | 0.070 |  | **1.413 (1.040-1.918)** | | **0.027** | |
|  | **180-day** |  |  |  |  |  |  | |  | |
| **MHNO** | 228 | 46 (20.2%) |  | Reference | |  | Reference | | | |
| **MUNO** | 170 | 47 (27.6%) |  | 1.476 (0.983-2.216) | 0.061 |  | 1.507 (0.993-2.289) | | 0.054 | |
| **MHO** | 848 | 158 (18.6%) |  | 0.909 (0.655-1.263) | 0.570 |  | 1.067 (0.757-1.504) | | 0.712 | |
| **MUO** | 972 | 273 (28.1%) |  | 1.470 (1.076-2.010) | 0.016 |  | **1.574 (1.141-2.170)** | | **0.006** | |
| The model was adjusted for age, gender, elective admission, primary payer, household income, patient discharge disposition, length of stay (LOS), total charges, emergency department records, transfer to rehabilitation care facilities, patient treatment location, local resident status, tobacco use, alcohol abuse, personal history of cancer chemotherapy, and overweight/obesity classification. | | | | | | | | | |  |

Supplementary Table 2. Readmission risk stratified by age and sex across three follow-up periods.

| **Variables** |  | **population** | **aHR (95% CI)** | **P value** |  | **population** | **aHR (95% CI)** | **P value** |  | **population** | **aHR (95% CI)** | **P value** |
| --- | --- | --- | --- | --- | --- | --- | --- | --- | --- | --- | --- | --- |
|  | **Age groups** | **120-day** | | |  | **150-day** | | |  | **180-day** | | |
| **MHNO** | ＜65 years | 147 | Reference | |  | 135 | Reference | |  | 123 | Reference | |
| **MUNO** |  | 47 | 1.625 (0.831-3.176) | 0.156 |  | 39 | 1.483 (0.710-3.097) | 0.294 |  | 31 | 1.789 (0.789-4.055) | 0.163 |
| **MHO** |  | 843 | 0.883 (0.578-1.351) | 0.567 |  | 746 | 0.882 (0.571-1.363) | 0.573 |  | 647 | 1.004 (0.627-1.608) | 0.986 |
| **MUO** |  | 539 | 1.524 (1.001-2.320) | 0.050 |  | **480** | **1.577 (1.028-2.418)** | **0.037** |  | **425** | **2.078 (1.310-3.296)** | **0.002** |
| **MHNO** | ≥ **65 years** | 133 | Reference | |  | 119 | Reference | |  | 105 | Reference | |
| **MUNO** |  | 184 | 1.470 (0.889-2.430) | 0.133 |  | 162 | 1.422 (0.854-2.368) | 0.176 |  | 139 | 1.430 (0.846-2.417) | 0.181 |
| **MHO** |  | 266 | 1.240 (0.759-2.024) | 0.391 |  | 229 | 1.307 (0.798-2.139) | 0.288 |  | 201 | 1.410 (0.857-2.319) | 0.176 |
| **MUO** |  | 720 | 1.369 (0.880-2.132) | 0.164 |  | 622 | 1.308 (0.835-2.048) | 0.241 |  | 547 | 1.272 (0.804-2.011) | 0.304 |
|  |  |  |  |  |  |  |  |  |  |  |  |  |
|  | **Sex** | **120-day** | | |  | **150-day** | | |  | **180-day** | | |
| **MHNO** | **Female** | 143 | Reference | |  | 128 | Reference | |  | 113 | Reference | |
| **MUNO** |  | 94 | 1.678 (0.933-3.018) | 0.084 |  | 84 | 1.636 (0.887-3.019) | 0.115 |  | 69 | 1.724 (0.902-3.292) | 0.099 |
| **MHO** |  | 788 | 1.185 (0.750-1.870) | 0.467 |  | 694 | 1.311 (0.812-2.117) | 0.268 |  | 604 | 1.439 (0.870-2.379) | 0.157 |
| **MUO** |  | **709** | **1.787 (1.145-2.789)** | **0.011** |  | **620** | **1.789 (1.125-2.845)** | **0.014** |  | **544** | **1.962 (1.206-3.193)** | **0.007** |
| **MHNO** | **Male** | 137 | Reference | |  | 126 | Reference | |  | 115 | Reference | |
| **MUNO** |  | 137 | 1.315 (0.790-2.189) | 0.293 |  | 117 | 1.290 (0.757-2.198) | 0.349 |  | 101 | 1.451 (0.827-2.545) | 0.194 |
| **MHO** |  | 321 | 0.788 (0.491-1.264) | 0.323 |  | 281 | 0.780 (0.481-1.266) | 0.315 |  | 244 | 0.859 (0.515-1.433) | 0.561 |
| **MUO** |  | 550 | 1.132 (0.744-1.721) | 0.563 |  | 482 | 1.189 (0.775-1.824) | 0.427 |  | 428 | 1.369 (0.874-2.145) | 0.171 |
|  | | | | | | | | | | | | |

Supplementary Table 3. LOS (days) and Total Charges ($) among different metabolic overweight/obesity phenotypes in six follow-up periods.

| **Variables** | **MHNO** | **MUNO** | **MHO** | **MUO** | **Total** | **P value** |
| --- | --- | --- | --- | --- | --- | --- |
| **30-day** |  |  |  |  |  |  |
| LOS(days)(mean(SD)) | 9.1(11.0)^a^ | **10.0(11.5)^a^** | 6.3(11.0)^b^ | 6.7(7.4)^b^ | 7.0(9.7) | **< 0.001** |
| LOS > 7 days | 146(38.3%)^a^ | **127(41.4%)^a^** | 299(20.3%)^b^ | 450(27.3%)^c^ | 1022(26.8%) | **< 0.001** |
| Total Charges($)(mean(SD)) | **130239.7 (235380.2)^a^** | 115530.9 (152621.2)^a,b^ | 90945.0 (151872.6)^b^ | 94755.7 (125064.2)^b^ | 98501.9 (152426.1) | **0.002** |
| Total Charges＞$50000 | 222(58.3%)^a^ | **180(58.6%)^a^** | 697(47.3%)^b^ | 870(52.8%)^a^ | 1969(51.7%) | **< 0.001** |
| **60-day** |  |  |  |  |  |  |
| LOS(days)(mean(SD)) | 8.7(9.6)^a^ | **9.8(11.1)^a^** | 6.2(8.9)^b^ | 6.6(7.3)^b^ | 6.9(8.6) | **< 0.001** |
| LOS > 7 days | 131(37.4%)^a^ | **116(41.1%)^a^** | 279(20.4%)^b^ | 406(26.9%)^c^ | 932(26.6%) | **< 0.001** |
| Total Charges($)(mean(SD)) | 122776.4 (216055.1)^a^ | 115135.9  (151195.4)^a^ | 91335.0 (154332.6)^a^ | 93613.7 (119637.8)^a^ | 97362.7 (148383.1) | **0.009** |
| Total Charges＞$50000 | 199(56.9%)^a^ | **165(58.5%)^a^** | 647(47.3%)^b^ | 787(52.1%)^a^ | 1798(51.2%) | **< 0.001** |
| **90-day** |  |  |  |  |  |  |
| LOS(days)(mean(SD)) | 8.9(9.9)^a^ | **9.8(11.5)^a^** | 6.1(8.6)^b^ | 6.6(7.3)^b^ | 6.9(8.6) | **< 0.001** |
| LOS > 7 days | 122(38.1%)^a^ | **101(39.6%)^a^** | 247(20.2%)^b^ | 378(27.2%)^c^ | 848(26.6%) | **< 0.001** |
| Total Charges($)(mean(SD)) | 125936.2 (224505.4)^a^ | 113372.0 (154030.3)^a,b^ | 89044.8 (151870.6)^b^ | 92532.7 (116800.1)^a,b^ | 96216.7 (147861.0) | **0.008** |
| Total Charges＞$50000 | **184(57.5%)^a^** | 143(56.1%)^a,b^ | 573(46.9%)^b^ | 721(51.9%)^a,b^ | 1621(50.9%) | **< 0.001** |
| **120-day** |  |  |  |  |  |  |
| LOS(days)(mean(SD)) | 8.9(9.6)^a^ | **9.8(11.7)^a^** | 6.0(8.3)^b^ | 6.7(7.5)^b^ | 6.9(8.5) | **< 0.001** |
| LOS > 7 days | 108(38.6%)^a^ | **91(39.4%)^a^** | 223(20.1%)^b^ | 346(27.5%)^c^ | 768(26.7%) | **< 0.001** |
| Total Charges($)(mean(SD)) | 120675.8 (164609.2)^a^ | **112616.7 (155060.1)^a,b^** | 87783.3 (135572.0)^b^ | 94187.8 (119858.4)^a,b^ | 95775.5 (134211.0) | **0.005** |
| Total Charges＞$50000 | 161(57.5%)^a^ | **130(56.3%)^a,b^** | 515(46.4%)^b^ | 658(52.3%)^a,b^ | 1464(50.9%) | **< 0.001** |
| **150-day** |  |  |  |  |  |  |
| LOS(days)(mean(SD)) | 9.0(9.7)^a^ | **9.9(10.7)^a^** | 5.9(7.5)^b^ | 6.7(7.3)^b^ | 6.8(8.1) | **< 0.001** |
| LOS > 7 days | 100(39.4%)^a^ | **82(40.8%)^a^** | 193(19.8%)^b^ | 306(27.8%)^c^ | 681(26.9%) | **< 0.001** |
| Total Charges($)(mean(SD)) | 122656.0 (168897.0)^a^ | 119930.0 (163823.1)^a,b^ | 87805.0 (137035.0)^b^ | 92618.4  (117931.0)^b^ | 95946.2 (135632.9) | **0.003** |
| Total Charges＞$50000 | 148(58.3%)^a^ | **118(58.7%)^a^** | 451(46.3%)^b^ | 569(51.6%)^a,b^ | 1286(50.8%) | **< 0.001** |
| **180-day** |  |  |  |  |  |  |
| LOS(days)(mean(SD)) | 9.3(10.1)^a^ | **9.5(9.9)^a^** | 5.9(7.4)^b^ | 6.6(7.2)^b^ | 6.8(7.9) | **< 0.001** |
| LOS > 7 days | **95(41.7%)^a^** | 68(40.0%)^a^ | 171(20.2%)^b^ | 267(27.5%)^c^ | 601(27.1%) | **< 0.001** |
| Total Charges($)(mean(SD)) | 127978.7 (176142.8)^a^ | **114518.8 (151778.8)^a,b^** | 88277.9 (139134.5)^b^ | 90984.9  (115070.9)^b^ | 95556.5 (135252.5) | **0.004** |
| Total Charges > $50000 | 134(58.8%)^a^ | **100(58.8%)^a^** | 396(46.7%)^b^ | 496(51.0%)^a,b^ | 1126(50.8%) | **0.001** |
|  | | | | | | |

Supplementary Table 4. ICD-10-CM codes used for inclusion, exclusion, and patient classification.

| **Diagnoses** | **ICD-10 Codes** |
| --- | --- |
| ITP | D693 |
| Underweight | Z681 |
| Normal body weight | Z6820, Z6821, Z6822, Z6823, Z6824 |
| Overweight | Z6825, Z6826, Z6827, Z6828, Z6829 |
| Obesity of grade 1 | Z6830, Z6831, Z6832, Z6833, Z6834 |
| Obesity of grade 2 | Z6835, Z6836, Z6837, Z6838, Z6839 |
| Obesity of grade 3 | Z6841, Z6842, Z6843, Z6844, Z6845 |
| Hyperlipidemia | E780-785 |
| Hypertension | G932, H35031, H35032, H35033, H35039, I10-13, I15, I16, I674, I973, R030 |
| Hyperglycemia | E08, E10, E11, E13, R7301, R7302, R7303 |
| Heart failure | I0981, I110, I130, I50, I9713 |
| Renal failure | N17, N19, N990, O0332, O0382, O0482, O0732, O084, O904, P960 |
| Coronary heart disease | I251 |
| Anemia | D460, D461, D462, D464, D5, D60, D61, D62, D63, D64, O9081, O990 |
| Neutropenia | D70 |
| Depression | F0631, F0632, F32, F33, F341 |
| Sepsis, unspecified organism | A419 |
| Pneumonia, unspecified organism | J189 |
| Acute kidney failure, unspecified | N179 |
| Hyp hrt & chr kdny dis w hrt fail and stg 1-4/unsp chr kdny | I130 |
| Hypertensive heart disease with heart failure | I110 |
| Non-ST elevation (NSTEMI) myocardial infarction | I214 |
| Morbid (severe) obesity due to excess calories | E6601 |
| Oth dis of the bld/bld-form org/immun mechnsm comp chldbrth | O9912 |
| Unilateral primary osteoarthritis, right knee | M1711 |
| Acute respiratory failure with hypoxia | J9601 |
